# The Velocity of Light Intensity Increase Modulates the Photoprotective Response in Coastal Diatoms

**DOI:** 10.1371/journal.pone.0103782

**Published:** 2014-08-01

**Authors:** Vasco Giovagnetti, Serena Flori, Ferdinando Tramontano, Johann Lavaud, Christophe Brunet

**Affiliations:** 1 Stazione Zoologica Anton Dohrn, Villa Comunale, Naples, Italy; 2 Littoral Environnement et Sociétés, Unité Mixte de Recherche 7266, CNRS-Université de La Rochelle, La Rochelle, France; Mount Allison University, Canada

## Abstract

In aquatic ecosystems, the superimposition of mixing events to the light diel cycle exposes phytoplankton to changes in the velocity of light intensity increase, from diurnal variations to faster mixing-related ones. This is particularly true in coastal waters, where diatoms are dominant. This study aims to investigate if coastal diatoms differently activate the photoprotective responses, xanthophyll cycle (XC) and non-photochemical fluorescence quenching (NPQ), to cope with predictable light diel cycle and unpredictable mixing-related light variations. We compared the effect of two fast light intensity increases (simulating mixing events) with that of a slower increase (corresponding to the light diel cycle) on the modulation of XC and NPQ in the planktonic coastal diatom *Pseudo-nitzschia multistriata*. During each light treatment, the photon flux density (PFD) progressively increased from darkness to five peaks, ranging from 100 to 650 µmol photons m^−2^ s^−1^. Our results show that the diel cycle-related PFD increase strongly activates XC through the enhancement of the carotenoid biosynthesis and induces a moderate and gradual NPQ formation over the light gradient. In contrast, during mixing-related PFD increases, XC is less activated, while higher NPQ rapidly develops at moderate PFD. We observe that together with the light intensity and its increase velocity, the saturation light for photosynthesis (Ek) is a key parameter in modulating photoprotection. We propose that the capacity to adequately regulate and actuate alternative photoprotective ‘safety valves’ in response to changing velocity of light intensity increase further enhances the photophysiological flexibility of diatoms. This might be an evolutionary outcome of diatom adaptation to turbulent marine ecosystems characterized by unpredictable mixing-related light changes over the light diel cycle.

## Introduction

Photosynthetic organisms have evolved a set of interconnected mechanisms of photoacclimation and photoprotection in order to efficiently regulate light harvesting, and prevent the impairment of photosynthesis and biomass production [1]. The non-photochemical fluorescence quenching (NPQ) is a photoregulative mechanism that rapidly and efficiently operates to mold photochemistry under changing light. NPQ dissipates excess light energy as heat and occurs in the light-harvesting complex antennae (LHC) of photosystem (PS) II [1]–[3]. Three major components are commonly identified in NPQ, on the basis of their different kinetics of formation and relaxation: the energy-dependent (qE), the state-transitions (qT), and the photoinhibitory (qI) quenching [1]–[3]. While the importance of each NPQ component varies among photosynthetic lineages, qE is essential for photoprotection in most of them and is mainly controlled by the build-up of a transthylakoidal proton gradient (ΔpH) and the inter-conversion between epoxidized and de-epoxidized forms of xanthophyll carotenoids during the so-called xanthophyll cycle (XC) [1], [3]–[5].

Several studies have demonstrated that the capacity of phytoplankton to efficiently regulate photosynthesis is functionally related to their adaptation to the underwater light environment [5]–[9]. Light fluctuations can indeed either limit the rate of photosynthesis (low light), or cause photo-oxidative stress due to the generation of reactive oxygen species in the photosynthetic apparatus (high light) [1]. Furthermore, when compared to terrestrial habitats, aquatic ecosystem mixing adds further unpredictability to light variations along the water column, which are either cyclic (i.e. diurnal/seasonal cycles) or irregular/stochastic (i.e. absorption and scattering due to dissolved substances and suspended particles in the water column, and intermittent cloud cover) [10], [11]. Cells therefore experience variations in the velocity of light intensity increase, from predictable diel cycle-related light changes to faster and unpredictable mixing-related ones. Changes in phytoplankton photophysiology have been observed during daylight in the field [12]–[14]. Moreover, major physiological processes and growth rate are differently affected in relation to the fluctuating light regimes tested and phytoplankton groups/species under investigation [15]–[17]. However, the effects of varying velocities of light intensity increase on phytoplankton capacity to photoprotect are unknown.

Among phytoplankton, diatoms constitute the most diversified group populating marine and freshwater ecosystems [18], [19], due to their plasticity to changing conditions, a feature that has been often related to their evolutionary origin [19]–[21]. Their ecological and biological success has largely influenced both the structure and biogeochemistry of contemporary oceans [20], where they contribute to approximately 40% of the oceanic primary production [19], [22]. Diatoms are known to have a remarkable capacity to cope with the variable underwater light environment [2], [3], [5]. They possess fucoxanthin (Fuco) chlorophyll (Chl) *a*/*c* binding proteins (FCP) as peripheral light-harvesting proteins and their antenna is organized in oligomeric complexes with groups−/species-dependent oligomeric state differences [2], [3].

NPQ in diatoms mainly relies on qE, that is triggered by (i) the light-dependent generation of a ΔpH, (ii) the presence of specific light-harvesting complex stress-related proteins (LhcSR), termed Lhcx, and (iii) the XC [2]–[4], [23]. In diatoms, qT seems to be missing [24], while the origin of qI – the most slowly forming and relaxing NPQ component that was originally ascribed to the photoinhibition of PSII reaction centre (RC) – is unclear, although the involvement of XC pigments is likely [2], [3].

High light induces the de-epoxidation of the epoxy-xanthophyll, diadinoxanthin (Dd), into the epoxy-free xanthophyll, diatoxanthin (Dt), while the epoxidation from Dt back to Dd occurs in low light or darkness [2]–[4]. Recently, it has been shown that the exposition to gradually increasing light intensities can result in a partial Dt epoxidation under moderate and high light in different Chl *a*/*c*-containing phytoplankton species [25]. Dt molecules are spatially and functionally segregated among several pools in the thylakoid membrane of diatoms [2], [3], [26], [27]. Under prolonged high light, Dd and Dt (as well as Lhcx proteins) can be *de novo* synthesized [2]–[4], [28], while Dt does not necessarily enhance NPQ [29], [30], but can fulfil an antioxidant function in the thylakoid membrane [27]. The violaxanthin (Vx) cycle, which is found in higher plants and green algae, is also present in diatoms, and consists of the de-epoxidation of Vx into zeaxanthin (Zx) via the intermediate xanthophyll, antheraxanthin (Ax), and reverse epoxidation [4], [31]. In diatoms, Vx serves as precursor pigment in the biosynthesis of Dd and their main FCP light-harvesting pigment, Fuco [31]–[33].

**Figure 1:**
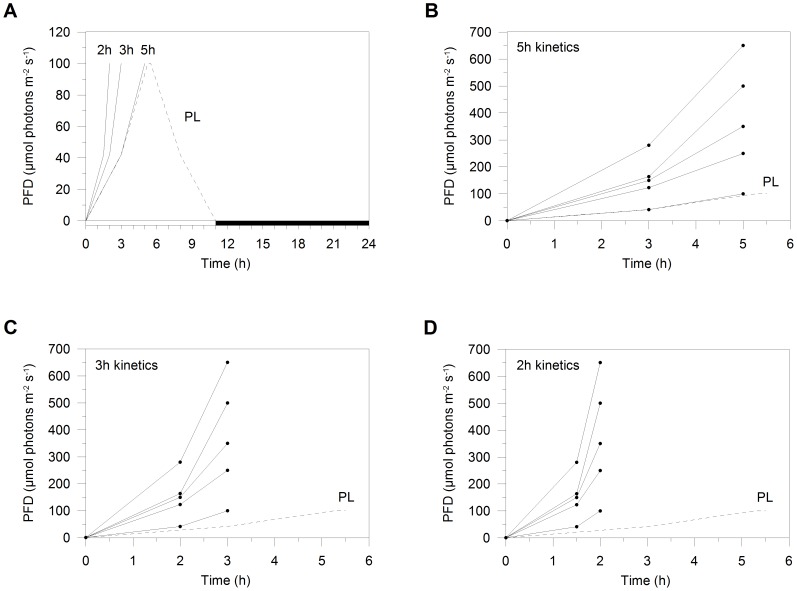
Preacclimation and experimental light conditions. (A) *Pseudo-nitzschia multistriata* cells were grown under a sinusoidal light regime set to peak at the PFD of 100 µmol photons m^−2^ s^−1^ (preacclimation light, PL; dashed line). After two weeks of preacclimation, cells in the exponential growth phase were shifted to three experimental light treatments, the 5 h (diel cycle-related PFD increase; B), 3 h and 2 h kinetics of light increase (mixing-related PFD increases; C and D, respectively), each characterized by light gradual increases peaking at the PFD of 100, 250, 350, 500 and 650 µmol photons m^−2^ s^−1^. In each panel, experimental light increases (solid lines) are compared to PL (dashed line). Triplicate samples were taken at three sampling time points during light increase (dots, B−D). Firstly, cultures were sampled in darkness. Then, after 3 h (5 h kinetics), 2 h (3 h kinetics), and 1.5 h (2 h kinetics), samples were taken at the PFD of 42, 123, 150, 164 and 280 µmol photons m^−2^ s^−1^ for the light condition peaking at 100, 250, 350, 500 and 650 µmol photons m^−2^ s^−1^, respectively. Lastly, cultures were sampled at PFD peaks.

The aim of our study is to investigate if the photoprotective mechanisms activated by coastal diatoms under an unpredictable and fast mixing-related photon flux density (PFD) increase differ from those in response to the predictable and slower diel cycle-related PFD increase. Here we address the effect of three velocities of light intensity increase on XC and NPQ modulation in the marine planktonic coastal diatom *Pseudo-nitzschia multistriata* (Takano) Takano, a toxic diatom known to form blooms in the Gulf of Naples (Mediterranean Sea) [34], [35], where it was isolated. *P. multistriata* photophysiological responses to each light kinetics were studied by subjecting cells to light intensities that progressively increased from darkness to five peaks, ranging from 100 to 650 µmol photons m^−2^ s^−1^ (Fig. 1).

Our results suggest that both the light intensity and the velocity of its increase control the regulation of photoprotection in *P. multistriata*. A slow light increase that resembles the light diel cycle enables a strong XC activation and moderate NPQ formation, through which cells can photoprotect against and photoacclimate to high light. Instead, velocities of light increase greater than that experienced during the light diel cycle lead to a flexible coupling between rapidly forming NPQ and XC operation, the former being enhanced, while the latter decreasing.

## Materials and Methods

### Ethics Statement

No specific permission was required for the isolation of the diatom *Pseudo-nitzschia multistriata* (strain SY416, Bacillariophyceae), which was carried out in the framework of the long-term ecological research MareChiara (LTER-MC, Stazione Zoologica Anton Dohrn, Naples, Italy), a research program conducted in coastal waters of the Gulf of Naples (Mediterranean Sea). No endangered or protected species has been used in this work.

### Culture Conditions

The coastal diatom *Pseudo-nitzschia multistriata* (Takano) Takano (strain SY416) was isolated (Gulf of Naples, Mediterranean Sea) and provided by SVM Tesson (Laboratory of Ecology and Evolution of Plankton, Stazione Zoologica Anton Dohrn, Naples, Italy). Cultures were grown non-axenically at 20°C in f/2 medium [36] made with locally obtained and sterilized seawater, using 225 cm^2^ polystyrene canted neck flasks (Corning Flask, Corning Inc., NY, USA). Cells were cultured under a sinusoidal light regime set to peak at the photon flux density (PFD) of 100 µmol photons m^−2^ s^−1^ (preacclimation light, PL), during two weeks before experiments, in a 11 hours (h) light/13 h dark photoperiod (Fig. 1A). Cells were gently and continuously flushed with sterile air, and maintained in exponential phase by daily and semi-continuous dilution. Temperature and pH were checked daily using an HI-9214-Stick pH meter (Hanna Instruments, Woonsocket, RI, USA). Light was provided using the Advanced Control Lighting System (ACLS) and Infinity XR4 pendant reflector (Aquarium Technologies, Sfiligoi S.r.l., Italy). Infinity XR4 was equipped with a HQI metal halide lamp (400 W, 10000 K). Photosynthetically available radiation (PAR) intensity was measured using a laboratory PAR 4 π sensor (QSL 2101, Biospherical Instruments, San Diego, CA, USA), while lamp spectral composition (PAR(*λ*)) was measured at light peak using a radiometer (Hyper OCR I, Satlantic, Halifax, CA).

### Experimental Design

After preacclimation (PL, Fig. 1A), *P. multistriata* cells in the exponential growth phase were shifted to the experimental light conditions before the light was switched on. Three experiments were performed in triplicate, testing three gradually increasing light treatments, namely the 5 h, 3 h and 2 h kinetics of light increase (Fig. 1B−D, respectively). During each of these three experimental kinetics of light increase, five light conditions were applied, characterized by light gradual increases peaking at the PFD of 100, 250, 350, 500 and 650 µmol photons m^−2^ s^−1^ (Fig. 1). Note that the 5 h kinetics of light increase peaking at 100 µmol photons m^−2^ s^−1^ was identical to PL (Fig. 1A−B). Samples were taken at three sampling time points during light increase (dots in Fig. 1B−D). Cultures were sampled 15 minutes (min) before light started to increase. Then, after 3 h (5 h kinetics), 2 h (3 h kinetics), and 1.5 h (2 h kinetics), samples were taken at the PFD of 42, 123, 150, 164 and 280 µmol photons m^−2^ s^−1^ for the light condition peaking at 100, 250, 350, 500 and 650 µmol photons m^−2^ s^−1^, respectively. The last sampling was carried out at the PFD peak (Fig. 1B−D). At each sampling time point, aliquots of 20–30 mL of culture were rapidly collected to measure Chl *a* fluorescence yield and non-photochemical fluorescence quenching (NPQ), and pigment content. Cell concentration and absorption spectrum were measured once a day during the first sampling time point.

### Cell Growth

During the preacclimation and the day in which each experiment was performed, growth was monitored by cell counting performed daily on triplicate sub-samples, using a Zeiss Axioskop 2 Plus microscope. Aliquots of 1 mL of algal culture were used to fill Sedgewick Rafter cell counting chambers. Growth rate was estimated from cell concentration measurements using the following equation, µ = ln [N_t2_/N_t1_]/[t_2_–t_1_], where µ is the growth rate (day^−1^) and N_t_ is the mean cell concentration at time t, and t_1_ and t_2_ correspond to the morning sampling times of days 1 and 2, respectively. The growth rate (µ) of *P. multistriata* cells grown under PL was 0.76±0.10 d^−1^ (*n* = 9, Table 1), and did not change during experiments, ranging between ∼0.68 and ∼0.90 (Table 1). Cell concentration ranged between ∼4.2 and ∼9.7×10^4^ cells mL^−1^, during preacclimation and experiments.

**Table 1:** Photosynthetic and physiological properties, and photosynthetic pigment content of *Pseudo-nitzschia multistriata*.

Parameters	Light conditions	Mean values ± SD
_rel_ETR_max_	Preacclimation	0.99±0.04
Ek	Preacclimation	246±12
µ	Preacclimation and 5 h, 3 h, 2 h kinetics	0.76±0.10
F_v_/F_m_	Preacclimation	0.71±0.01
Chl *a* cell^−1^	5 h, 3 h, 2 h kinetics	4.63±1.14
Chl *c*_1_ Chl *a* ^−1^	5 h, 3 h, 2 h kinetics	3.97±0.77
Chl *c*_2_ Chl *a* ^−1^	5 h, 3 h, 2 h kinetics	6.46±0.94
Chl *c*_3_ Chl *a* ^−1^	5 h, 3 h, 2 h kinetics	7.41±2.01
Fuco Chl *a* ^−1^	5 h, 3 h, 2 h kinetics	67.63±6.75

The measurement of photosynthetic and physiological properties was performed on cells in the exponential growth phase, during preacclimation, the day before the experiments started. The growth rate did not change during experiments. _rel_ETR_max_, maximal relative electron transport rate (in mol e^−^ g Chl *a* ^−1^ h^−1^); Ek, saturation light for photosynthesis (in µmol photons m^−2^ s^−1^); µ, growth rate (in d^−1^); F_v_/F_m_, photosystem II maximal photochemical efficiency. Values are means ± SD (*n* = 9). Chlorophyll *a* cellular content (Chl *a*, in 10^−16^ mol Chl *a* cell^−1^) and photosynthetic accessory pigments Chl *a* ^−1^ content (in mol pigment/100 mol Chl *a*) measurements were performed during experiments. Fuco, fucoxanthin: Chl *c*, chlorophyll *c*_1_,_ 2_,_ 3_. Pigment data are means ± SD of the all data set (*n* = 135).

### Pigment Analysis

High performance liquid chromatography (HPLC) was performed to analyse pigment content. Aliquots of 10 mL of algal culture were filtered onto GF/F glass-fibre filters (Whatman, Maidstone, UK) and immediately stored in liquid nitrogen until further analysis. Triplicate samples were taken during each sampling time point. Pigments were extracted by mechanical grinding during 3 min in 2 mL of a 100% methanol solution. Then, the homogenate was filtered onto Whatman 25 mm GF/F glass-fibre filters (Whatman, Maidstone, UK) and the volume of the extract was accurately measured. Prior to injection into the loop of the HPLC system, 250 µL of an Ion Pairing Agent (ammonium acetate 1 mol L^−1^, final concentration 0.33 mol L^−1^) were added to 0.5 mL of the pigment extract and incubated for 5 min in darkness at 4°C. This extract was then injected in the 50 µL loop of the Hewlett Packard series 1100 HPLC system (Hewlett Packard, Wilmington, NC, USA), equipped with a reversed-phase column (2.6 µm diameter C_8_ Kinetex column, 50 mm×4.6 mm; Phenomenex, USA). The temperature of the column was steadily maintained at 20°C and the flow rate of the mobile phase was set up at 1.7 mL min^−1^. The mobile phase was composed of eluent A, a solvent mixtures of methanol and aqueous ammonium acetate (70/30, v/v), while eluent B was methanol. During a 12 min-lasting elution, the gradient between the solvents was programmed: 75% A (0 min), 50% A (1 min), 0% A (8 min), 0% A (11 min), 75% A (12 min). Pigments were detected spectrophotometrically at 440 nm using a model DAD, Series 1100 Hewlett-Packard photodiode array detector. Fluorescent pigments were detected in a Hewlett-Packard standard FLD cell series 1100, with excitation and emission wavelengths set at 407 and 665 nm, respectively. For determination and quantification of pigments, calibration curves were obtained using pigment standards from Danish Hydraulic Institute (DHI) Water & Environment (Hørsholm, Denmark).

### Absorption Spectrum

Aliquots of 10 mL of algal culture were filtered onto Whatman GF/F filters (Whatman, Maidstone, UK) and immediately frozen. Absorption spectrum measurements were performed as previously described, and correction factors (e.g. due to filter absorption enhancement) were applied accordingly [37]. Absorption was measured between 280 and 800 nm with 1-nm increments on a spectrophotometer (Hewlett-Packard HP-8453E) equipped with an integrating sphere RSA-HP-53 (Labsphere Inc., North Sutton, NH, USA). The mean integrated absorption value (*a* ^*^) was thus normalized by the chlorophyll (Chl) *a* concentration to obtain the Chl *a*-specific absorption coefficient (*a* ^*^_ph_; m^2^ mg Chl *a* ^−1^). The number of absorbed photons Chl *a* ^−1^ integrated over time (expressed in mol photons mg Chl *a* ^−1^) was calculated as the product of PAR (*λ*, 400–700 nm) and *a* ^*^_ph_ (*λ*, 400–700 nm) integrated over the time course of the experiments.

### Chl *a* Fluorescence Yield and Non-Photochemical Fluorescence Quenching (NPQ)

Photochemical efficiency of photosystem (PS) II was estimated by pulse amplitude fluorescence (PAM) measurements, using a PHYTO-PAM fluorometer (Heinz Walz, Effeltrich, Germany). F_0_ and F_m_ are defined as the minimum PSII fluorescence yield and the maximum PSII fluorescence yield measured on 15 min dark-acclimated cells, while being termed F_0_′ and F_m_′ when measured on light-acclimated cells. F_m_ or F_m_′ were measured after a saturating pulse of red light (2400 µmol photons m^−2^ s^−1^, lasting 450 ms), causing a complete reduction of the PSII acceptor pool. The maximum photosynthetic efficiency of PSII is calculated as the ratio F_v_/F_m_, where F_v_ is the variable fluorescence emission and is equal to F_m_−F_0_.

The electron transport rate (ETR) *versus* irradiance (E) curves were performed on 15 min dark-acclimated samples by applying 10 stepwise increasing actinic irradiances (E, from 1 to 1500 µmol photons m^−2^ s^−1^), at intervals of 2 min each. The maximal relative rate of linear electron transport, normalized by Chl *a* concentration (_rel_ETR_max_, expressed in mol e^−^ g Chl *a* ^−1^ h^−1^), was calculated as _rel_ETR_max_ = (F_v_′/F_m_′)×PFD×(*a* ^*^_ph_/2), where F_v_′ and F_m_′ are PSII variable and maximal fluorescence yield, respectively, for illuminated cells (measured at the end of the 2 min lasting actinic light), and PFD is the incident irradiance (expressed in µmol photons m^−2^ s^−1^). The Chl *a*-specific absorption coefficient *a* ^*^_ph_ (see above) was divided by two, assuming that the excitation energy is evenly distributed between the two photosystems. The photosynthetic parameters, maximal relative electron transport rate (_rel_ETR_max_) and saturation light for photosynthesis (Ek) were retrieved from the ETR-E curves [38].

Non-photochemical fluorescence quenching (NPQ) was measured on 15 min dark-acclimated cells. Actinic light was fixed at 480 µmol photons m^−2^ s^−1^ and the cells were illuminated for 10 min, and the maximum fluorescence yield was estimated each min. Actinic light intensity during the measurement was chosen in order to saturate photosynthesis in control cultures and ensure maximal NPQ amplitude. NPQ was quantified by the ‘Stern-Volmer’ expression, NPQ = (F_m_/F_m_′) –1, where F_m_′ is the maximum PSII fluorescence yield of light-acclimated cells [39].

A sustained light-acclimated NPQ (NPQ_sl_) was calculated as (F_mt0_/F_m_′) –1 [40]. F_mt0_ corresponds to F_m_ measured from the dark-acclimated cells sampled during the first sampling time point. F_m_′ is measured at each sampling time point on light-acclimated cells. Differently from the sustained phase of NPQ (NPQ_s_) estimated in [40], NPQ_sl_ represents the overall NPQ, i.e. the fraction that rapidly relaxes and its more sustained components, totally accumulating during the light increase.

### Statistical Analysis

Student’s *t*-test analysis for comparison of means and Spearman correlation were performed using the software Statistica (StatSoft, OK, USA).

## Results and Discussion

### Photoacclimation to gradual increases of PFD

Growth rate (µ), photosystem (PS) II maximal photochemical efficiency (F_v_/F_m_), and maximal relative electron transport rate (_rel_ETR_max_; see Table 1) confirmed the healthy physiological state of *P. multistriata* cells grown under preacclimation light (PL, i.e. sinusoidal light peaking at the PFD of 100 µmol photons m^−2^ s^−1^; Fig. 1A). From the measured _rel_ETR_max_ (0.99±0.04 mol e^−^ g Chl *a* ^−1^ h^−1^), an oxygen evolution rate of ∼250 µmol O_2_ mg Chl *a* ^−1^ h^−1^ was estimated. The saturation light for photosynthesis (Ek) was ∼250 µmol photons m^−2^ s^−1^ in *P. multistriata* cells under PL (Table 1). This means that the Ek value was higher than the maximal PFD reached during preacclimation, probably indicating that this species could not decrease Ek to values below ∼250 µmol photons m^−2^ s^−1^ when subjected to PL. Interestingly, similar results have been reported on three different Chl *a*/*c*-containing species (belonging to the class of Bacillariophyceae [9] and Pinguiophyceae [41]), grown under the same light conditions provided by the same light system in this study. It should be noted that the light system we applied mainly provides blue wavelengths, which are known to be more efficiently used by diatoms than red or green wavelengths [42], [43].

For each kinetics of light increase, our experimental design allowed us to test the photophysiological regulation of *P. multistriata* under three increasing light conditions that reached PFD peaks higher than Ek and two conditions reaching PFD peaks lower than or similar to Ek. Whatever was the condition of light increase, and regardless of the kinetics of light increase, the Chl *a* cellular content and photosynthetic pigment Chl *a* ^−1^ content did not change significantly over time (p>0.05, *n* = 15; Table 1), with concentrations of Chl *a* significantly correlated to those of Fuco, Chl *c*_1_, *c*_2_, and *c*_3_ (p<0.005, *n* = 45). Fuco was the main accessory pigment, with its pool size being approximately ten- and seventeen-fold higher than that of Chl *c*_2_/*c*_3_ and *c*_1_, respectively (Table 1). The presence of Chl *c*_3_, which is a pigment rarely found in diatoms, agrees with previous findings on the same species ([42] and references therein).

The absence of a photoacclimative response involving variation in the photosynthetic pigment content contrasts with the results generally observed in previous studies (e.g., [44]–[46]). Some authors [46] showed that the exposure to high light (500 µmol photons m^−2^ s^−1^) of *Phaeodactylum tricornutum* cells caused a rapid down-regulation of Chl *a* biosynthesis and transcripts encoding putative light harvesting antenna proteins, as well as an immediate decline in Fuco cellular content and the subsequent decrease in Chl *a* and *c* cellular content. The reason of such a difference with our results is linked to the gradual light increase applied in our study, in contrast to the sudden light increase that is often applied (e.g., [46]). Indeed, the use of an abrupt light increase activates regulative and photoacclimative strategies related to a stress-response, which might involve a prompt rearrangement of the light harvesting system and consequent decrease in photosynthetic pigment pool size, together with XC/NPQ induction (e.g., [45], [46]). In contrast, a “naturally occurring” gradual increase of light allows cells to progressively modulate the photoprotective process. In this framework, the modulation of XC not only acts as short-term photoprotective process controlling NPQ formation, but also enables cells to photoacclimate to gradual increases of light without significantly changing the light-harvesting capacity of the photosynthetic antenna. This confirms previous results obtained in a study conducted on different Chl *a*/*c*-containing species [25], in which the authors also show that the epoxidation of Dt to Dd can take place under moderate and high light in some species when cells undergo a gradual light increase. Overall, these results are a further proof that the experience of a gradual light increase enables cells to efficiently regulate their photophysiological properties by properly balancing photoacclimation and photoprotection.

However, results on photoacclimation and photoprotection regulation should be considered in the context of light adaptation [25] and nutrient availability ([47], [48] and references therein). Indeed, Dimier et al. [25] showed different photoresponses to PFD increase in high light-, low light- and variable light-adapted phytoplankton species, such as the coastal diatom *P. multistriata*, on the basis of their XC characteristics. Since it is known that light history influences photoregulation [8], it should be underlined that preacclimation light (PL, 100 µmol photons m^−2^ s^−1^; Fig. 1) corresponds to PAR values measured at a depth range of 7–12 m in the mixed layer of the coastal waters of the Gulf of Naples (Mediterranean Sea; Brunet, unpublished data).

Furthermore, nutrient supply controls phytoplankton cellular response in the field, modifying the balance between light-harvesting processes and those that generate and utilize energy sources (adenosine 5′-triphosphate, ATP, and reduced nicotinamide adenine dinucleotide phosphate, NADPH), hence modulating cell photoacclimation/photoprotection dynamics [47], [48]. Therefore, our results refer to nutrient-replete conditions, such as those found during the onset of the spring bloom.

**Figure 2:**
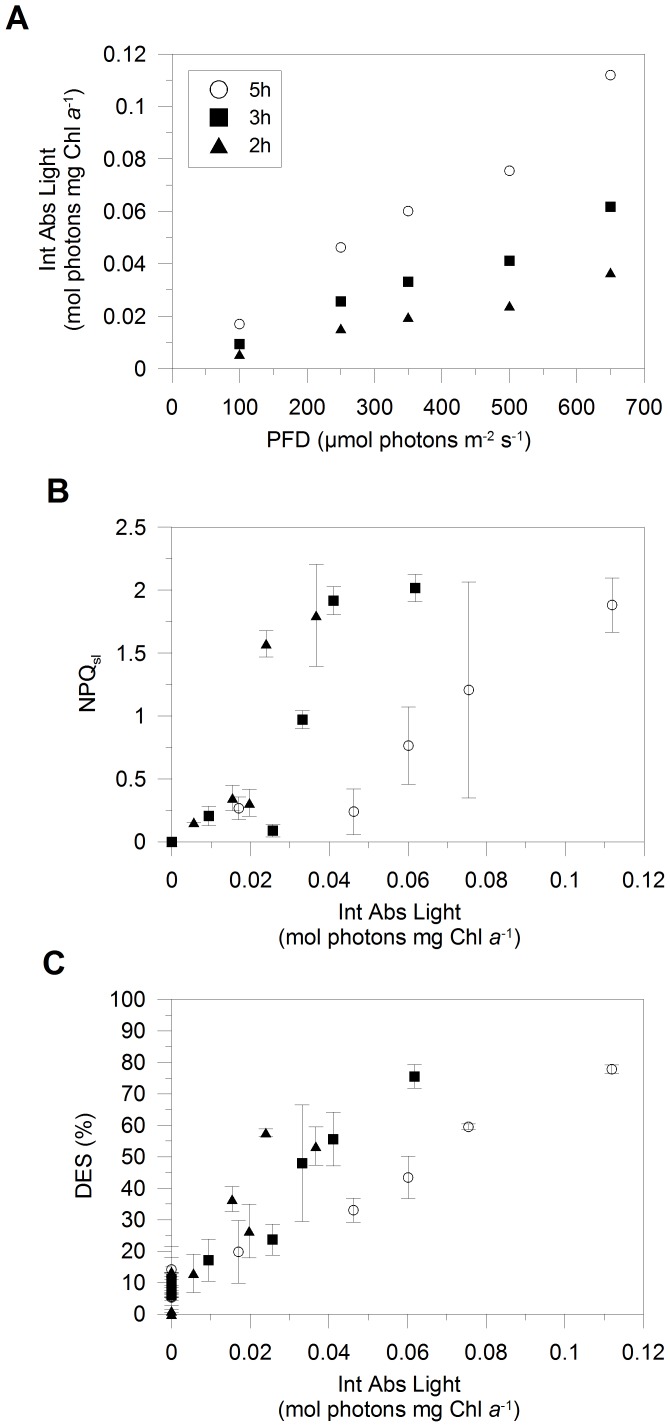
Influence of the kinetics of light increase on the photoprotection modulation. (A) Evolution of the number of absorbed photons per Chl *a* integrated over time (integrated absorbed light, Int Abs Light; expressed in mol photons mg Chl *a* ^−1^) over the light gradient, at the PFD peaks of 100, 250, 350, 500 and 650 µmol photons m^−2^ s^−1^, during the 5 h (white dots), 3 h (black squares) and 2 h kinetics of light increase (black triangles). Induction of the sustained light-acclimated NPQ (NPQ_sl_; B) and evolution of the de-epoxidation state (DES = Dt/[Dd+Dt]; C) *versus* Int Abs Light during the 5 h (white dots), 3 h (black squares) and 2 h kinetics of light increase (black triangles). Values are means ± SD (*n* = 3).

One of the main aspects addressed by this study is the role played by light increase velocity on the photoregulation capacity of *P. multistriata*. Figure 2A depicts the number of absorbed photons Chl *a* ^−1^ integrated over time that characterizes the three tested light treatments, simulating diel cycle-related (5 h kinetics) and mixing-related PFD increase conditions (3 h and 2 h kinetics; Fig. 1). Over the gradient of the time-integrated absorbed photons per Chl *a*, the faster kinetics of light increase (3 h and 2 h kinetics) distinctively affect the sustained light-acclimated NPQ (NPQ_sl_, which is the overall NPQ that totally accumulates during the light increase; Fig. 2B) and the de-epoxidation state (DES = Dt/[Dd+Dt]; Fig. 2C), when compared to the slowest condition (5 h kinetics). These results reveal that changes in the kinetics of light increase influence XC/NPQ modulation (see next subsections), thus probably impacting the productivity of the mixed layer.

### XC and NPQ responses to a diel cycle-related PFD increase

**Figure 3:**
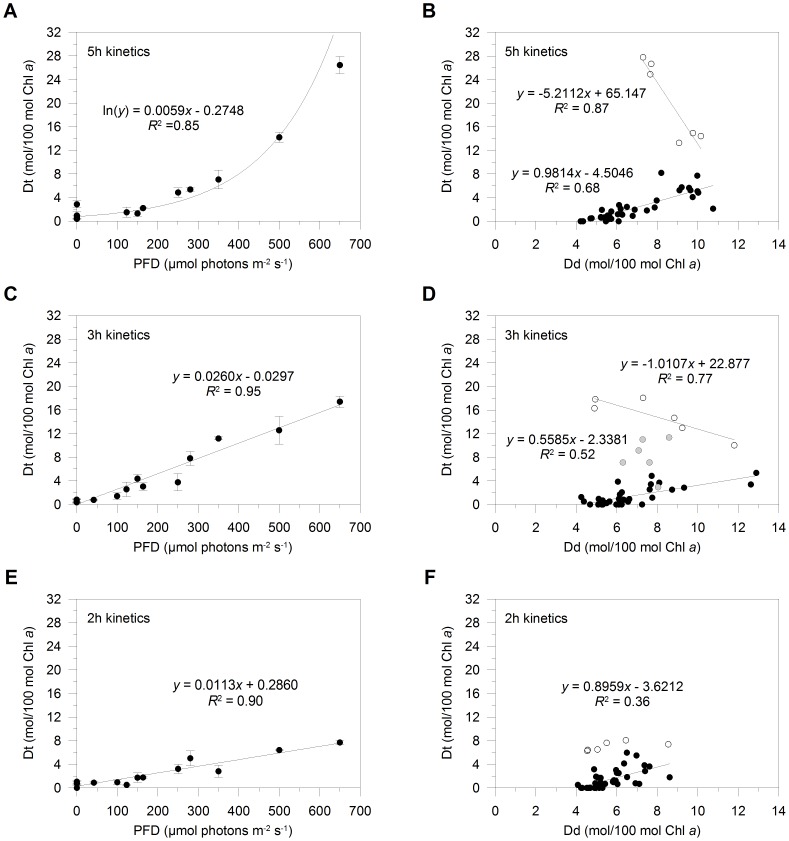
Xanthophyll cycle modulation. Evolution of diatoxanthin (Dt)/chlorophyll (Chl) *a* (in mol Dt/100 mol Chl *a*) over the light gradient, in *Pseudo-nitzschia multistriata* cells experiencing light gradual increases peaking at the PFD of 100, 250, 350, 500 and 650 µmol photons m^−2^ s^−1^, during the 5 h (A), 3 h (C) and 2 h kinetics of light increase (E). Values are means ± SD (*n* = 3). Relationship between Dt and diadinoxanthin (Dd)/Chl *a* (in mol pigment/100 mol Chl *a*), during the 5 h (B), 3 h (D) and 2 h kinetics of light increase (F). In (B) and (F) data measured at PFD ≤350 µmol photons m^−2^ s^−1^ (black dots, *n* = 39) and ≥500 µmol photons m^−2^ s^−1^ (white dots, *n* = 6) are discerned. In (D) data measured at PFD ≤250 µmol photons m^−2^ s^−1^ (black dots, *n* = 33), at 280 and 350 µmol photons m^−2^ s^−1^ (grey dots, *n* = 6), and at PFD ≥500 µmol photons m^−2^ s^−1^ (white dots, *n* = 6) are discerned.

In the 5 h kinetics of light increase, Dt synthesis exponentially increased over the light range and Dt Chl *a* ^−1^ reached the highest value measured among the tested light treatments (26.5±1.4 mol Dt/100 mol Chl *a*; Fig. 3A). The augment in Dt pool size largely relied on Dd *de novo* synthesis as revealed by the significant and positive correlation between Dd and Dt when PFD was ≤350 µmol photons m^−2^ s^−1^ (*R* ^2^ = 0.68, p<0.005, *n* = 39; black dots in Fig. 3B). In contrast, when PFD was ≥500 µmol photons m^−2^ s^−1^, the relationship between the two xanthophylls was inverse (*R* ^2^ = 0.87, p<0.025, *n* = 6; white dots in Fig. 3B), showing a further (almost three-fold) increase in Dt pool size through Dd pool depletion.

DES linearly increased over the light gradient (*R* ^2^ = 0.92, p<0.005) reaching the maximal value of 78%. The strong activation of the XC in *P. multistriata* is fostered by an efficient enhancement of the carotenoid biosynthetic pathway, as demonstrated by the significant correlation found between either Vx or Zx Chl *a* ^−1^ and Dt Chl *a* ^−1^ (when Vx cycle xanthophylls were detected, *R* ^2^ = 0.48, p<0.01, *n* = 25, and *R* ^2^ = 0.59, p<0.05, *n* = 9, respectively; Fig. S1A and S1B), as well as between β carotene (β-Car) and Dd Chl *a* ^−1^ (*R* ^2^ = 0.52, p<0.005, *n* = 44; Fig. S2A). These results further confirm the role of Vx cycle pigments as biosynthesis precursors of Dd and Fuco [31], [33], a feature that has been regarded as metabolically advantageous in order to poise photoprotection and light harvesting in Chl *a*/*c*-containing phytoplankton groups [25], [31], [33]. Indeed, while Ax was not found in our study [31], both Vx and Zx were detected all along the light range (Table 2), with their pool size especially increasing as PFD was ≥280 µmol photons m^−2^ s^−1^ (Table 2), i.e. close to the Ek value (Table 1). This finding makes Ek a key parameter in controlling the photoprotective response development at the pigment content level, and not only the limiting/optimal light switch for photosynthesis.

**Table 2:** Carotenoid content of *Pseudo-nitzschia multistriata* cells.

Pigments		5 h kinetics	3 h kinetics	2 h kinetics
β-Car Chl *a* ^−1^	All data	4.17±0.83 (*n* = 44)	4.56±0.60 (*n* = 45)	4.71±0.65 (*n* = 44)
Vx Chl *a* ^−1^	<280 µmol photons m^−2^ s^−1^	0.34±0.04 (*n* = 15)	0.31±0.09 (*n* = 14)	0.45±0.08 (*n* = 6)
Vx Chl *a* ^−1^	≥280 µmol photons m^−2^ s^−1^	0.53±0.14 (*n* = 10)	0.33±0.08 (*n* = 10)	0.45±0.09 (*n* = 3)
Zx Chl *a* ^−1^	<280 µmol photons m^−2^ s^−1^	0.36±0.11 (*n* = 3)	0.28±0.08 (*n* = 13)	0.00
Zx Chl *a* ^−1^	≥280 µmol photons m^−2^ s^−1^	0.46±0.16 (*n* = 6)	0.44±0.20 (*n* = 7)	0.44±0.03 (*n* = 3)

β-carotene (β-Car), violaxanthin (Vx) and zeaxanthin (Zx)/chlorophyll (Chl) *a* (in mol pigment/100 mol Chl *a*) of *Pseudo-nitzschia multistriata* cells experiencing light gradual increases peaking at the PFD of 100, 250, 350, 500 and 650 µmol photons m^−2^ s^−1^, during the 5 h, 3 h and 2 h kinetics of light increase (see Fig. 1B−D). Pigment values are means ± SD.

**Figure 4:**
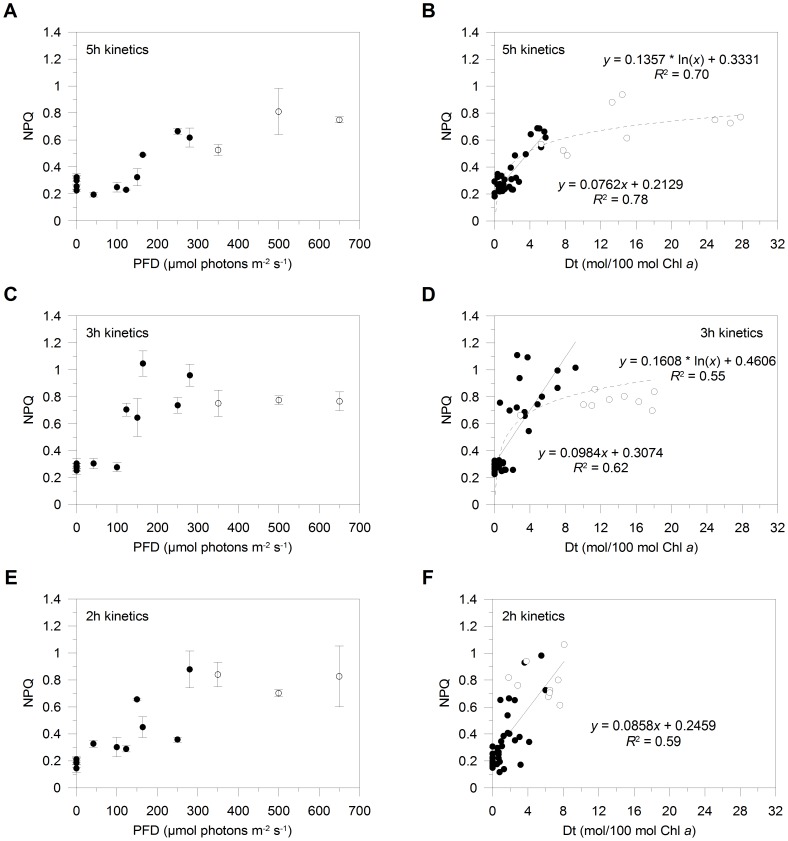
Non-photochemical fluorescence quenching (NPQ), and relationship between NPQ formation and diatoxanthin (Dt) synthesis. Induction of NPQ over the light gradient in *Pseudo-nitzschia multistriata* cells experiencing light gradual increases peaking at the PFD of 100, 250, 350, 500 and 650 µmol photons m^−2^ s^−1^, during the 5 h (A), 3 h (C) and 2 h kinetics of light increase (E). Values are means ± SD (*n* = 3). Relationship (*n* = 45) between NPQ and Dt Chl *a* ^−1^ (in mol Dt/100 mol Chl *a*) in *P. multistriata* cells during the 5 h (B), 3 h (D) and 2 h kinetics of light increase (F). Black and white dots are data measured at PFD ≤280 and ≥350 µmol photons m^−2^ s^−1^, respectively.

Despite cells activated the strongest Dt synthesis in this condition (Fig. 3A), NPQ was the lowest among light treatments (Fig. 4A). NPQ gradually increased over the light gradient and reached the maximal value of 0.81±0.17 at 500 µmol photons m^−2^ s^−1^, after which it remained stable (Fig. 4A). This suggests that the Dt pool size synthesized to cope with a diurnal light increase is not entirely involved in NPQ formation, as also reported in other diatom species [26], [29], [30]. Although NPQ was weakly induced, its development was significantly correlated to Dt Chl *a* ^−1^ (*R* ^2^ = 0.70, *n* = 45, p<0.005; Fig. 4B) and DES (*R* ^2^ = 0.65, *n* = 45, p<0.005). Intriguingly, the linear relationship between NPQ formation and Dt synthesis, which is commonly reported (e.g., [29], [49], [50]), was only found for PFD ≤ Ek (precisely for PFD ≤280 µmol photons m^−2^ s^−1^, *R* ^2^ = 0.78, *n* = 36, p<0.005; black dots in Fig. 4B). For PFD greater than Ek, NPQ only slightly increased and poorly relied on the further synthesis of Dt (white dots in Fig. 4B). Such a discrepancy in the expected Dt/NPQ linear relationship might be related to the spatial and functional heterogeneity of Dt pools in the thylakoid membrane of diatoms [26], [27], [51]. Dt molecules might be located among the monogalactosyl-diacylglycerol (MGDG) molecules of the lipid shield that surrounds the FCPs, instead of being bound to FCP specific antenna polypeptides [27], [28], [51]. These Dt molecules are likely to prevent lipid peroxidation [27] instead of effectively participating to NPQ, which needs the so-called ‘activation’ of Dt molecules through the protonation of some FCP binding sites during the ΔpH build-up (Δ522 nm fingerprint) [26], [49], [52]. The weak development of NPQ is also in line with the fact that Dt molecules dissolved in MGDG shield are not able to interact excitonically with Chl *a* [27], hence decreasing the light energy that is channelled to the PSII RC.

**Figure 5:**
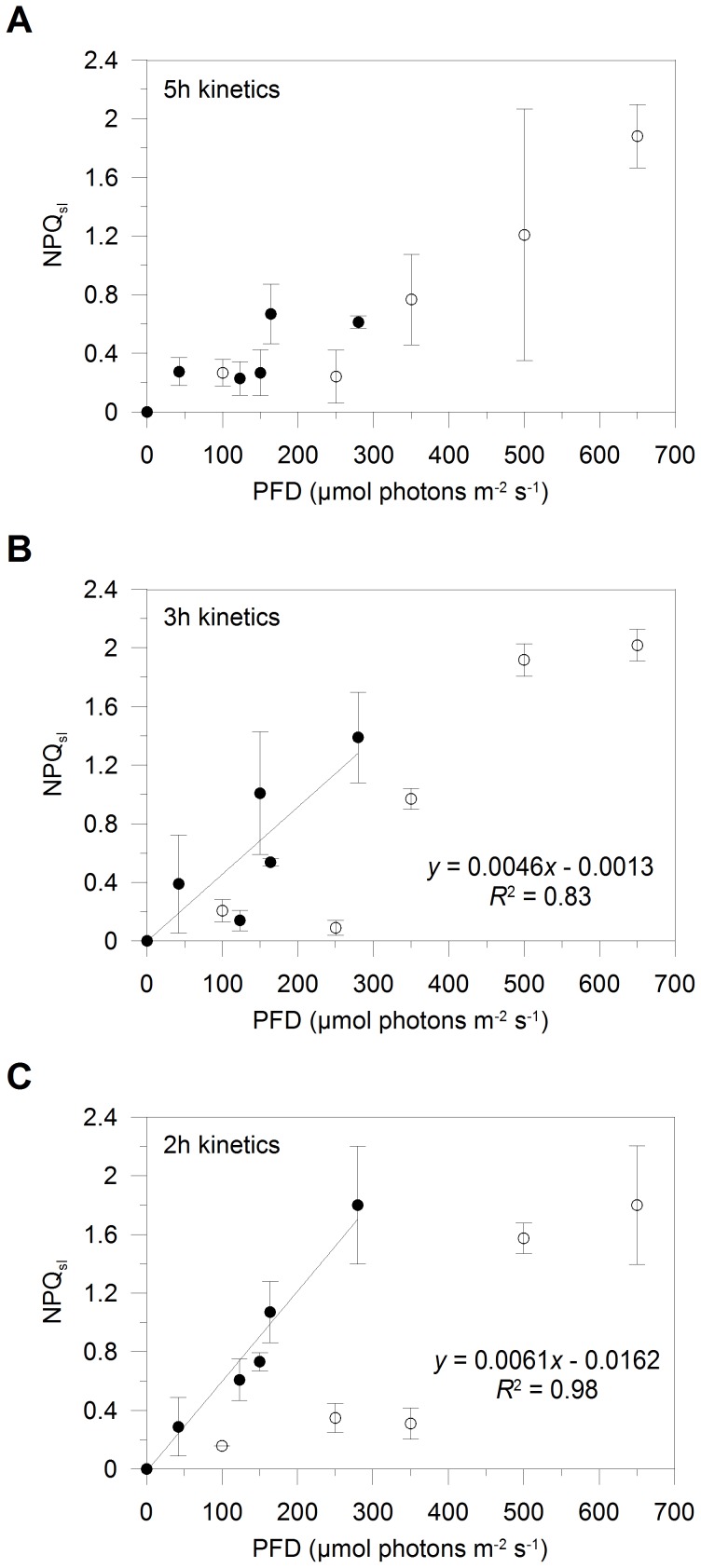
Sustained light-acclimated non-photochemical fluorescence quenching (NPQ_sl_). Induction of NPQ_sl_ over the light gradient, in *Pseudo-nitzschia multistriata* cells experiencing light gradual increases peaking at the PFD of 100, 250, 350, 500, and 650 µmol photons m^−2^ s^−1^, during the 5 h (A), 3 h (B) and 2 h kinetics of light increase (C). Black dots are values estimated for the first and second sampling time point, white dots are values estimated for the last sampling time point. Values are means ± SD (*n* = 3).

NPQ_sl_ almost gradually increased over the light range and reached its maximal value at 650 µmol photons m^−2^ s^−1^ (1.9±0.2, Fig. 5A). Differently from what we observed in the two faster kinetics of light increase (see below), NPQ_sl_ values were low and quite stable for PFD ≤ Ek, while increasing more steeply when PFD was>Ek in the 5 h kinetics (Fig. 5A).

### XC and NPQ response to mixing-related PFD increase

The faster was the kinetics of light increase, the less activated was the XC (Fig. 3C and E), leading to a decrease of the maximal Dt Chl *a* ^−1^ from ∼27 mol Dt/100 mol Chl *a* (5 h kinetics, Fig. 3A) to 17.4±1.0 (3 h kinetics, Fig. 3C) and 7.7±0.4 mol Dt/100 mol Chl *a* (2 h kinetics, Fig. 3E). Dt synthesis linearly increased (p<0.005) during the two fast kinetics of light increase, in a stronger manner in the 3 h than the 2 h kinetics (Fig. 3C and E). These results demonstrate that XC operation is not exclusively driven by the light intensity increase, but also by the velocity at which cells undergo such an increase of light intensity. It is interesting to note that light intensity and velocity of its increase affect XC modulation in opposite ways.

During the 3 h kinetics, Dt and Dd Chl *a* ^−1^ were positively correlated for PFD ≤ Ek (until 250 µmol photons m^−2^ s^−1^, *R* ^2^ = 0.52, p<0.005, *n* = 33; black dots in Fig. 3D). For PFD similar to or greater than Ek (at 280 and 350 µmol photons m^−2^ s^−1^; grey dots in Fig. 3D), no correlation was observed between the two xanthophylls and the Dt pool size further increased through Dd pool depletion (at 280 µmol photons m^−2^ s^−1^) and subsequent Dd *de novo* synthesis (until 500 µmol photons m^−2^ s^−1^). The decrease in Dd Chl *a* ^−1^ at 280 µmol photons m^−2^ s^−1^ indicates that the rate of Dd de-epoxidation was faster than that of its replenishment, consistently with a greater requirement of Dt synthesis at PFD∼Ek (see below). When PFD was ≥500 µmol photons m^−2^ s^−1^, Dt and Dd Chl *a* ^−1^ were instead negatively correlated, revealing that Dt pool size continued to increase again by depleting the Dd pool (*R* ^2^ = 0.77, p<0.05, *n* = 6; white dots in Fig. 3D).

During the 2 h kinetics, the increase in Dd Chl *a* ^−1^ was the least strong among the tested light kinetics (Fig. 3F), which might in part explain the weakest Dt synthesis in this condition (Fig. 3E). Dt and Dd Chl *a* ^−1^ were positively correlated until PFD was ≤350 µmol photons m^−2^ s^−1^ (*R* ^2^ = 0.36, p<0.025, *n* = 39; black dots in Fig. 3F). When PFD became ≥500 µmol photons m^−2^ s^−1^, Dt pool size slightly increased without correlating to Dd pool variations (white dots in Fig. 3F).

Taken together, these results reveal that XC operation is light increase kinetics-dependent and its efficiency decreases when the velocity of light intensity increase is too fast. Furthermore, they highlight a new feature of XC functioning: even though XC is rapidly activated in response to light changes, it seems to be best fitted to cope with slow light increases, as the case of the light diel cycle or low mixing. This feature probably relates to the time needed to activate the carotenoid biosynthetic pathway for XC pigment pool replenishment. Indeed, during both mixing-related PFD increases, Vx Chl *a* ^−1^ was lower than the values measured in the 5 h kinetics and stable regardless of the light intensity (Table 2), with no correlation between Vx and Dt Chl *a* ^−1^ (Fig. S1C and S1E). Whereas in the 3 h kinetics Zx Chl *a* ^−1^ mean values were similar to those found in the 5 h kinetics (Table 2) and a significant correlation was found between Zx and Dt Chl *a* ^−1^ (when Zx was detected, *R* ^2^ = 0.56, p<0.01, *n* = 20; Fig. S1D), Zx was almost never detected in the 2 h kinetics (Table 2 and Fig. S1F). Concomitantly, β-Car Chl *a* ^−1^ was quite stable among light treatments (Table 2), and β-Car and Dd Chl *a* ^−1^ were not correlated in both mixing-related conditions (Fig. S2B and S2C), in contrast to what we observed during the diel cycle-related one (Fig. S2A).

Although XC is not efficiently activated in cells subjected to light increases faster than the predictable light diel cycle, mixing-related PFD increases enhance NPQ, when compared to the diel cycle-related one (Fig. 4). Intriguingly, we measured NPQ maxima at moderate PFD.

During the 3 h kinetics, NPQ most steeply increased until PFD reached Ek (Fig. 4C) and relied on a rapid and strong Dt synthesis (*R* ^2^ = 0.62, p<0.005, *n* = 36; black dots in Fig. 4D). This is demonstrated by the almost two-fold greater amount of Dt (up to ∼9 mol Dt/100 mol Chl *a* at 280 µmol photons m^−2^ s^−1^; Fig. 4D) than that measured during the 5 h kinetics (up to ∼5 mol Dt/100 mol Chl *a*; Fig. 4B), which relied on the greatest Dd Chl *a* ^−1^ value at 250 µmol photons m^−2^ s^−1^ (up to ∼13 mol Dd/100 mol Chl *a*) and Dd depletion at 280 µmol photons m^−2^ s^−1^ (Fig. 3D). When PFD was >280 µmol photons m^−2^ s^−1^, NPQ was instead lower and stable, despite Dt content almost doubled (up to ∼20 mol Dt/100 mol Chl *a*; white dots in Fig. 4D). In this condition, an enhanced synthesis of Dt molecules that functionally participate to NPQ [52], [53] might relate to the harsher build-up of ΔpH caused by the greater PFD change per unit time than during the light diel cycle. Thus, a prompt and efficient regulation of XC functionally drives a rapid NPQ formation, despite the lower accumulation of Dt molecules than during a diel cycle-related PFD increase. These results also indicate that the fastest and strongest NPQ induction serves as first photoprotective defense to cope with a rapid increase of light. Therefore, *P. multistriata* cells are able to modulate the functional link between NPQ formation and XC operation in relation to light intensity and velocity of its increase probably via the intensity-dependent electron transport rate (ETR) and the coupled transthylakoidal proton gradient [26], [54].

During the 2 h kinetics, the relationship between NPQ and Dt Chl *a* ^−1^ was linear (*R* ^2^ = 0.59, p<0.005, *n* = 45; Fig. 4F) and did not change over the full range of PFD, in contrast to the 5 h and 3 h kinetics (Fig. 4B and D). The highest NPQ was measured for PFD ≥ Ek, i.e. when PFD becomes saturating for photosynthesis, although these NPQ values were lower than those measured in the 3 h kinetics. These evidences suggest that a very fast light intensity increase, as the case of the 2 h kinetics, is too rapid for an efficient modulation of NPQ and XC in *P. multistriata* cells.

In contrast to the 5 h kinetics, in the two mixing-related PFD increases, both light intensity and time affected the NPQ_sl_ dynamics over the light gradient (Fig. 5B and C). In the 3 h kinetics, until PFD was ∼Ek, NPQ_sl_ was higher when similar PFD values were reached more rapidly (<2 h, black dots *versus* 3 h, white dots; Fig. 5B). Same results were obtained in the 2 h kinetics (compare ≤1.5 h, black dots *versus* 2 h, white dots; Fig. 5C). A strong difference between the three experiments also concerned the value of NPQ_sl_ developed in the faster response when PFD was ∼Ek: 0.61±0.04, 1.39±0.31 and 1.80±0.40 in the 5 h, 3 h and 2 h kinetics, respectively (black dots in Fig. 5). We might therefore speculate that NPQ components independent of Dt activation and rapidly induced are more developed the faster is the mixing-related increase of light (i.e. in the 2 h than in the 3 h kinetics), to compensate the impaired Dt synthesis. Diatoms can indeed develop a diverse set of mechanisms of Dt-independent NPQ, such as the PSII electron transfer cycle [7], [55], the conformational changes in the core of PSII [56], and the aggregation of FCPs functionally-detached from PSII [51], [57], [58]. The capacity to form functionally disconnected FCP complexes can partially explain the degree of amplification of the Dt-dependent quenching among different diatom species and strains [26]. Interestingly, NPQ_sl_ values measured at 500 and 650 µmol photons m^−2^ s^−1^ were almost similar among the three kinetics of light increase (∼1.9, Fig. 5). This result emphasizes the fact that the overall NPQ (i.e. NPQ_sl_) is enhanced when cells experience mixing events until they reach a photosynthesis-saturating PFD, above which similar NPQ_sl_ values are developed regardless of time. Below the photosynthesis-saturating PFD, the NPQ/XC coupling is strongly dependent on the velocity of the light intensity increase through interactions between ETR, the ΔpH build-up, the lumen pH-dependent activation of the Dd de-epoxidase and ‘activation’ of Dt molecules in the NPQ process [26], [54]. Once diatoms establish the NPQ component that is triggered by the fast net accumulation of Dt, the breakdown of the proton gradient does not lead to its direct relaxation, which rather depends on the efficiency of the epoxidation of Dt to Dd [49] and removal of Dt from its FCP-binding sites [3]. Since mixing seems to complementarily activate Dt-dependent and Dt-independent NPQ components possibly characterized by different kinetics of induction and relaxation, we might hypothesize that their interplay is crucial to dissipate excess light energy and modulate diatom photosynthesis in the mixed layer.

### Conclusions

Our results show that during a diel cycle-related PFD increase, a strong and prolonged activation of XC is the main photoprotective response developed by the diatom *P. multistriata*. XC operation triggers gradual NPQ formation and strong accumulation of Dt molecules over the light range, through an effective regulation of the carotenoid biosynthesis that involves changes in β-Car and Vx cycle xanthophyll pool size. In this condition, the photosynthetic machinery is able to progressively acclimate to the diurnal light increase and balance all photosynthetic regulatory partners, thus preventing a strong NPQ formation. The weak development of NPQ also highlights the photoprotective efficiency of the synthesis of Dt in coping with a predictable diel cycle-related PFD increase. In contrast, mixing-related velocities of light increase favour NPQ development, and do not allow an efficient XC activation. Indeed, the carotenoid biosynthetic pathway is only partially activated under mixing regimes, causing a limited synthesis of Vx cycle xanthophylls. In case of mixing events, Dt-independent NPQ components seem to be more induced to compensate the impairment of the Dt synthesis. This flexible coupling between NPQ and XC in relation to predictable/unpredictable changes in light environment fits with the outstanding photophysiological plasticity of diatoms, possibly reflecting an evolutionary adaptation they acquired thriving in turbulent waters.

During the applied gradual light increases, we found the highest development of NPQ at moderate light, i.e. when PFD becomes saturating for photosynthesis. Moreover, the whole photoprotective response is activated before cells undergo light conditions that saturate photosynthesis. These results therefore suggest that the saturation light for photosynthesis (Ek) plays a relevant role on the modulation of the photoprotective processes, XC and NPQ, together with the velocity of light increase.

This study gives new insights into the role of water mixing on the photophysiology of coastal diatoms and the importance of NPQ formation/XC operation in coping with light variability. Furthermore, it highlights the necessity of conducting experiments in which phytoplankton are submitted to gradual light increase conditions, in order to gain a better understanding of their ecophysiological plasticity in the field, which in turn might improve mathematical models of phytoplankton growth and succession [59]–[61].

## Supporting Information

Figure S1 **Violaxanthin (Vx) cycle xanthophylls** ***versus*** **diatoxanthin (Dt) amount.** Relationship between Vx and Dt/chlorophyll (Chl) *a* (in mol pigment/100 mol Chl *a*), and between zeaxanthin (Zx) and Dt/Chl *a* (in mol pigment/100 mol Chl *a*) in *Pseudo-nitzschia multistriata* cells experiencing light gradual increases peaking at the PFD of 100, 250, 350, 500 and 650 µmol photons m^−2^ s^−1^, during the 5 h (A and B), 3 h (C and D) and 2 h kinetics of light increase (E and F).
(TIF)

Figure S2 **β-carotene (β-Car)** ***versus*** **diadinoxanthin (Dd) amount.** Relationship between β-Car and Dd/chlorophyll (Chl) *a* (in mol pigment/100 mol Chl *a*, *n* = 45) in *Pseudo-nitzschia multistriata* cells experiencing light gradual increases peaking at the PFD of 100, 250, 350, 500 and 650 µmol photons m^−2^ s^−1^, during the 5 h (A), 3 h (B) and 2 h kinetics of light increase (C).
(TIF)
