# Supplementary figures and images for: The Velocity of Light Intensity Increase Modulates the Photoprotective Response in Coastal Diatoms
Source: PLoS One. 2014 Aug 1;9(8):e103782. doi: 10.1371/journal.pone.0103782 (PMC4118909; doi:10.1371/journal.pone.0103782)

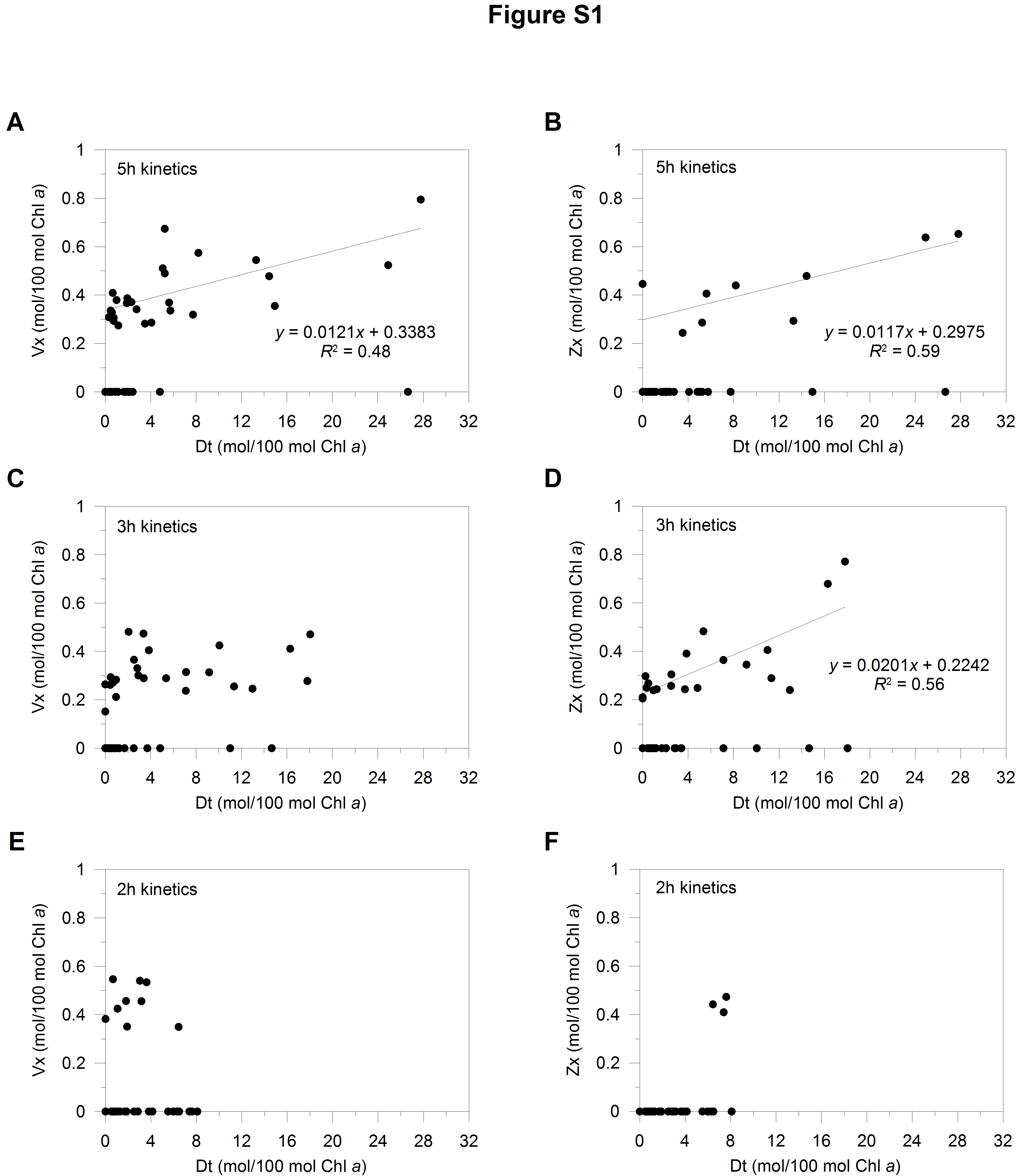

Supplement: Figure S1 — Violaxanthin (Vx) cycle xanthophylls versus diatoxanthin (Dt) amount. Relationship between Vx and Dt/chlorophyll (Chl) a (in mol pigment/100 mol Chl a), and between zeaxanthin (Zx) and Dt/Chl a (in mol pigment/100 mol Chl a) in Pseudo-nitzschia multistriata cells experiencing light gradual increases peaking at the PFD of 100, 250, 350, 500 and 650 µmol photons m−2 s−1, during the 5 h (A and B), 3 h (C and D) and 2 h kinetics of light increase (E and F). (TIF) [file pone.0103782.s001.tif]

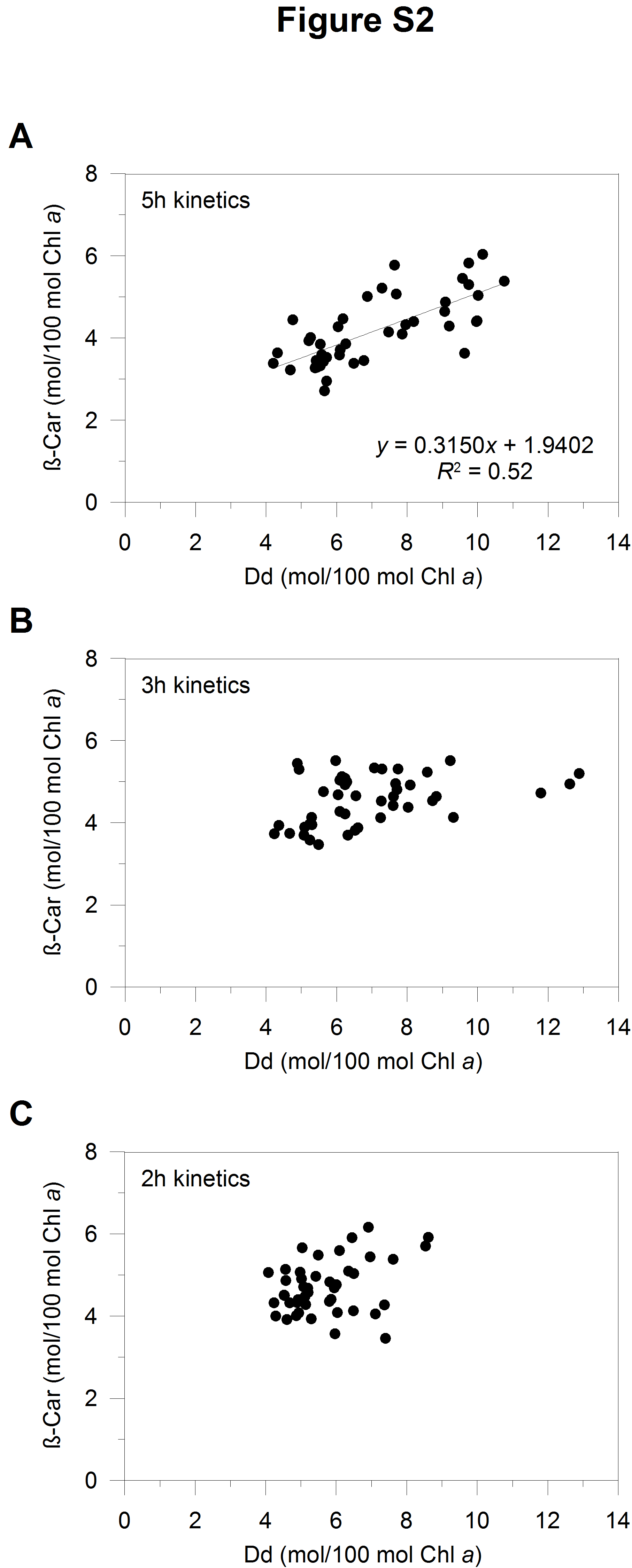

Supplement: Figure S2 — β-carotene (β-Car) versus diadinoxanthin (Dd) amount. Relationship between β-Car and Dd/chlorophyll (Chl) a (in mol pigment/100 mol Chl a, n = 45) in Pseudo-nitzschia multistriata cells experiencing light gradual increases peaking at the PFD of 100, 250, 350, 500 and 650 µmol photons m−2 s−1, during the 5 h (A), 3 h (B) and 2 h kinetics of light increase (C). (TIF) [file pone.0103782.s002.tif]
